# Manual Therapy: The Historical, Current, and Future Role in the Treatment of Pain

**DOI:** 10.1100/tsw.2007.14

**Published:** 2007-02-02

**Authors:** A. Russell Smith

**Affiliations:** Department of Athletic Training and Physical Therapy, Brooks College of Health, University of North Florida, Jacksonville, USA

**Keywords:** manual therapy, manipulation, massage, spinal, extremity, massage, rehabilitation

## Abstract

Manual therapy has been an approach in the management of patients with various disorders dating back to ancient times and continues to play a significant role in current health care. The future role of manual therapy in health care is an important area of research. This paper reviews the history of manual therapy, examines the current literature related to the use of manual techniques (including manipulation, massage, and nerve manipulation), and discusses future research topics. The literature related to manual therapy has historically been anecdotal and theoretical, and current research tends to have a generic approach with broad definitions of manual therapy and inconsistencies in the classification of specific disorders. Systematic reviews of various types of manual therapy have differed on their conclusions regarding the effectiveness of this treatment modality. The current demand in health care for evidence-based practice necessitates a movement towards more specificity in the research of the effectiveness of manual therapy, with emphasis on specific patient signs and symptoms and specific manual techniques that result in effective care.

## INTRODUCTION

Manual therapy is the use of the hands to apply a force with a therapeutic intent. Manual therapy approaches and techniques include massage, joint mobilization/manipulation, myofascial release, nerve manipulation, strain/counterstrain, and acupressure. Historically, manual therapy has had an important role in health care that continues today. The future role of manual therapy in health care is uncertain. Identification of the efficacy and effectiveness of specific manual therapy approaches and techniques with specific patient characteristics is needed. Individuals within various health care professions practice different forms of manual therapy to address patients with various types of pain and/or functional limitations, contributing to the confusion regarding the appropriate mode and delivery of this intervention. This paper will review the history of manual therapy from ancient times to the present, examine the current literature related to the use of manual techniques (including manipulation, massage, and nerve manipulation), and future research topics.

## HISTORY

Historically, manual therapy has been an approach in the management of patients with various disorders dating back to ancient times[1,2,3]. The laying on of hands to heal is well documented in the Old Testament and was supported by Hippocrates in the 5th century BC. Hippocrates recommended the use of manual therapy including prone traction with associated spinal manipulation. He demonstrated the effectiveness of various manual techniques and suggested modifications in the delivery of force parameters, such as direction, speed, and frequency of the manipulations including high-velocity thrust techniques[1,2,3]. In the 2nd century AD, Galen applied manual medicine to the extremities and spine including massage and joint manipulation. Abu’Ali ibn Sina (980–1037), an Arabian physician, authored an authoritative text on medicine including the role of manual medicine. The Renaissance surgeon Ambrose Pare recommended the treatment of thoracic kyphosis to include traction with the use of ropes and boards, and manual pressure over the vertebra. Manual treatment was further developed in Europe by generations of “bonesetters”, manual practitioners who passed on the art of technique from one family member to another. The bonesetter’s approach grew as medically trained practitioners moved away from movement and emphasized pharmacological approaches. Sir Herbert Barker (1869–1950), a famous bonesetter from Great Britain, expressed his dismay in his autobiography that his contemporary medical practitioners ignored the manual techniques for patients with pain and stubbornly declined help from the bonesetters in spite of the evidence of the effectiveness of manual treatment[1,2,3].

In the U.S., the late 19th century found the beginnings of osteopathy and chiropractic[1,2,4]. Andrew Taylor Still, unhappy with orthodox medical practice, founded osteopathy in 1874 with emphasis on holistic health care including preventative medicine and the vital role of the musculoskeletal system in health. Still proposed the interrelationship between the body’s structure and function, and the body’s innate ability to heal. He then proposed that this interrelationship could be enhanced through manual techniques, specifically osteopathic manipulative treatment. In 1895, Daniel David Palmer founded chiropractic, emphasizing the “The Law of the Nerve”. Palmer proposed that a vertebra may become subluxed, impinging structures in the intervertebral foramen and impairing innervated structures, resulting in a diseased condition. He further proposed that manual adjustment (manipulation) of the subluxation would thereby restore health to the affected tissues.

Physical therapy, existing under other names earlier in other countries in Europe, was formally founded in 1899 in England and established in 1921 in the U.S.[1,2,4]. Manipulation of joint and soft tissues was a foundational skill for physical therapists, rehabilitating patients with a variety of disorders. Medical physician James Mennell taught physical therapists the techniques of manipulation in the early 1900s in England, as did the British physician James Cyriax in the mid-1900s. Both Mennell and Cyriax integrated manual therapy into orthopedic medicine, emphasizing the importance of mechanical diagnosis and conservative treatment of musculoskeletal disorders

## PURPOSE

Currently, manual therapy is used in the treatment of patients with various disorders including extremity joint dysfunction[5,6,7], spinal dysfunction[1,2,8,9,10], lymphadema[4], TMJ disorders[11], headaches[12,13,14,15], cystic fibrosis[16,17], nerve entrapments[5], and following immobilization[6,7]. Different schools of thought emphasize different goals of intervention and actual techniques. Techniques essentially vary according to the goal, the tissue emphasized, and force parameters applied during the intervention. Goals of manual therapy include increasing joint movement quantity and quality, promoting stability, decreasing pain, promoting nerve tension and mobility, and improving function. Practitioners apply manual techniques to affect various tissues including joints, nerves, muscles, bones, and fascia. Practitioners of manual therapy may vary their delivery of the force to the patient including force direction, duration, contact, frequency, and/or velocity. Additionally, the force may be generated externally or internally, by the therapist or by the patient, respectively. Although various schools of thought in manual therapy propose that one parameter may be more beneficial than others, there is no evidence to support such claims. The velocity of the technique has been the subject of great debate. The merits of high-velocity techniques (thrust techniques) have been compared to low-velocity techniques (nonthrust techniques). Some have described high-velocity techniques as *manipulation* and low-velocity techniques as *mobilization*[18], while others consider these terms as synonymous[19]. Generally, the distinction places greater emphasis on the velocity of the technique as compared to other parameters. For the purpose of this paper, the latter view will be followed, and the terms mobilization and manipulation will be used interchangeably. This is consistent with the definition for manipulation/mobilization in the Guide to Physical Therapist Practice[19]: “A manual therapy technique comprised of a continuum of skilled passive movement to the joints and/or related soft tissues that are applied at varying speeds and amplitudes, including a small amplitude high velocity therapeutic movement.”

## EVIDENCE

The literature related to manual therapy has historically been anecdotal and theoretical. More recently, increased reports of clinical trials related to manual therapy have been observed in the literature[21,22,23,24,25,26,27,28,29,30,31,32,33,34,35,36,37]. Current research into the effectiveness of manual therapy has a general approach with broad definitions of the actual intervention and inconsistencies in the classification of specific disorders that the manual intervention is directed towards. Interpretations of the results of the investigations vary. The following will review the evidence related to the more common techniques of joint manipulation/mobilization, massage, and nerve mobilization, emphasizing the application of these techniques to patients with various disorders.

### Joint Manipulation

Systematic reviews of joint manipulation have differed on their conclusions regarding the effectiveness of this treatment modality. While several reviews conclude that spinal manipulation is helpful as intervention for patients with spinal disorders[8,9,21,22,23,18,27,28], others report that the preponderance of poor-quality studies result in inconclusive decisions[24,25,29,30,32]. Still other systematic reviews[26,33,34] conclude that spinal manipulation is not an effective treatment modality.

This lack of a consistent view of the role of spinal manipulation is also observed on review of national clinical practice guidelines[38,39,40,41,42]. Spinal manipulation is recommended for patients with acute low back pain (LBP) in several national clinical practice guidelines: U.S.[35,37], New Zealand[38], Denmark[39], and Finland[40]. However, spinal manipulation is not recommended for patients with acute LBP in clinical practice guidelines in the Netherlands[41] and Australia[42]. Several factors may contribute to this disparity of opinion.

A primary issue in these mixed results is the heterogeneity of the patient populations and the conditions being examined. These conflicting results and conclusions relative to the role of spinal manipulation may be due to a generic approach towards experimental groups that actually require more individualized approaches[43,44]. Additionally, manipulation has often been treated as one modality, when in reality, the force parameters may vary greatly. LBP is the result of multiple contributing factors that vary from patient to patient. Researchers who expose all patients with general complaints of LBP to the same type of intervention are making the false assumption that this group is homogeneous. Patients with LBP may have underlying mobility, stability, or combination impairments. Pathology may also differ within the subjects grouped under the umbrella term LBP. One experimental approach that could prove more valid is the examination of subgroups of patients that are more homogeneous.

In 1998, the Forum for Primary Care Research on Low Back Pain[45] proposed the identification of these subgroups as their number one research priority. Identifying these subgroups according to pathoanatomical mechanisms has been futile[43,44], and current emphasis is on identifying subgroups according to patient history and physical examination findings[46,47,48,49,50,51,52,53,54,55]. In addition to identifying a more homogenous group of patients with LBP that may benefit from manipulation, a more specific presentation of the type of manipulation performed is needed. Clarification of the various parameters of the actual technique may identify particular techniques that may be more beneficial with a specific subgroup of patients. Recently, clinical prediction rules have been formulated to address this need for explicit definitions of technique and back pain[53,54]. Flynn et al. have identified five factors as predictors of success in manipulation interventions for patients with LBP[54]. Patients meeting four of five of the following criteria were most likely to benefit within 1 week with a maximum of two manipulation interventions: symptom duration less than 16 days, no symptoms distal to knee, score less than 19 on the Fear-Avoidance Belief Questionnaire (FABQ), at least 1 hypomobile lumbar segment, and at least one hip with greater than 35 degrees of internal rotation. The clinical prediction rule was further validated in a multicenter randomized control trial[53] that randomly assigned 131 patients to receive exercise alone or manipulation and exercise for 4 weeks by a physical therapist. The effectiveness of the spinal manipulation was found to be greatest for the patients who were positive on at least four of the five criteria. The results of this study emphasize the importance of matching individual patients to specific interventions most likely to be beneficial.

The role of joint manipulation in the management of headaches has met similar conflicting reviews in the literature[56,57,58]. Several randomized clinical trials (RCTs) demonstrate that manipulation (where) is beneficial in the management of patients with cervicogenic headaches[12,13,14,56]. In their systematic review, Astin and Ernst[57] reported that definitive conclusions were not possible due to the small number of published investigations and the high degree of sample heterogeneity,. They identified that a common problem throughout the RCTs was that trial designs made it difficult to distinguish the specific source of the improvements observed following manipulation.

Cervical manipulation is often used to treat head and neck pain and movement disorders[5,56,57]. The results are equivocal regarding the benefits of cervical manipulation when considering the risk-benefit ratios[58,59,60]. Due to potential risks for harm to neurovascular structures[66,67,68,69], several have questioned the use of thrust manipulation to the cervical spine[60,70]. Several studies have demonstrated that the velocity of manipulation is not a factor in the effectiveness of a manipulation technique as nonthrust, and thrust techniques have been shown to be similarly beneficial[61,62]. Other studies have proposed that thrust manipulation is no more effective than other forms of treatment including placebo[7,56,58,63,64,65]. Although the exact risk is impossible to determine, the risk of serious complications following cervical manipulation has been estimated as approximately six per 10 million[71], a figure that assumes that only one in ten serious incidents is reported in the literature. Review of the potential risk with cervical manipulation must be considered in light of other risks in health care. On the other hand, cervical manipulation is considered to have less risk than the risk of serious complications associated with use of NSAIDS[59], and of cervical spine surgery, which has a 700 times greater risk of serious complication[72].

Manual therapy is also often applied to extremity disorders, particularly in the presence of limited mobility[5,6,7,73,74,75,76,77,78,79]. Several investigations have found that manipulation of the extremities may improve movement and decrease pain, but again the evidence is equivocal and more research is indicated[7,78,79,80,81,82,83,84,85]. The role of manual therapy for patients with carpal tunnel syndrome has been supported[78,79]. Manipulation of the carpal bones, combined with a manual stretch of the flexor retinaculum in a single cohort study, was found to decrease pain[78] and improve wrist extension range of motion. The role of manipulation following immobilization for fractures of extremity joints has also been investigated[6,7,80,81,82,83,84,85]. Manipulation as a component of rehabilitation post-Colles fracture casting was found in one investigation (without a control group) to result in increased wrist extension and improved pain[6], however, no clinical difference was found in two other studies between treatment and control groups[81,82]. Joint mobilization combined with exercise in patients with metacarpophalangeal fractures was demonstrated to be more effective in improving mobility than exercise alone[80]. In contrast, an investigation, albeit low sample size (12), of the role of manipulation in the rehabilitation following ankle fractures demonstrated no statistical difference between joint mobilization combined with whirlpool and exercise as compared to whirlpool and exercise alone[83].

Two studies investigating the role of manipulation following acute ankle sprain reported significant improvement in patient function and pain following ankle manipulation as compared to the more traditional treatment approach of rest, ice, compression, and elevation treatment strategy[84,85]. Improvements included increased ankle range of motion, decreased pain, quicker return to activities of daily living, and increased quality of gait[84]. Additionally, the manipulation group demonstrated a decreased number of treatment sessions as compared with the more typical treatment. A repeated-measures, double-blind randomized controlled trial investigated the immediate effects of manipulation (specifically, manipulation with movement) on patients with subacute ankle sprain[85]. Following the manipulation technique, patients demonstrated a significant increase in weight-bearing ankle dorsiflexion range of motion, however, no change was observed in pressure and thermal pain thresholds. The authors proposed that the manipulation effect is more a mechanical rather than hypoalgesic effect in subacute ankle sprains.

Combining manipulation techniques with therapeutic exercises appears to be more beneficial than exercise alone in patients with subacromial impingement syndrome[86,87]. In a multisite RCT, 52 participants with subacromial impingement syndrome were randomly assigned to one of two groups: supervised exercise alone or supervised exercise with mobilization of shoulder, cervical, and upper thoracic regions[86]. Participants received six 30-min treatments over 6 weeks. At 1-month follow-up, the group receiving mobilization had significantly less pain, with resisted movements and improved strength, as compared to the exercise group alone. At the 2-month follow-up, significant improvements were reported in function and pain by the mobilization group.

Manual therapy has varying degrees of support in the management of patients with extremity pain and dysfunction. Specifically, the role of manual therapy for patients with carpal tunnel syndrome has been supported. Weak evidence exists relative to the role of manual therapy with restricted extremity joints postimmobilization. Manipulation after ankle sprains has been supported.

## Massage

Massage has been a method of treatment for centuries and is gaining popularity as an alternative and complimentary approach in health care. Investigations into the effect of massage are limited. Massage has a proposed effect on pain level, blood flow, flexibility, and various psychological parameters[88,89]. The research on the effect of massage on blood flow indicates no effect on large arterial blood flow[90,91], however, the blood flow in small vessels is noted to increase with massage[92]. Massage has also been reported to decrease the intensity of delayed-onset muscle soreness (DOMS) in several investigations[93,94,95,96]. A recent investigation by Zainnudin et al.[97] demonstrated that massage was effective in decreasing the severity of DOMS (VAS) as compared to no treatment. Additionally, reduced swelling was noted following massage with no change in muscle function.

Massage has been demonstrated to have a positive psychological effect[89] including improving general mood[17,98] and decreasing anxiety[88], stress[99] and depression[100]. Massage, by parents and physical therapists, has also been shown to affect positively pulmonary function in children with cystic fibrosis and asthma[16,98]. Massage to children with cystic fibrosis performed by the parent improved the mood and decreased anxiety in children and their parent[17]. A meta-analysis of 37 RCTs[88] concluded that single applications of massage lowered anxiety, blood pressure, and heart rate, however, no change in immediate pain reports was noted.

In contrast to the above effects, the European Guidelines[101] reported that massage is not supported in the literature to be more effective than sham procedures in the treatment of LBP. The European Guidelines concluded that they could not recommend massage in the management of patients with chronic LBP. In 2001, the Philadelphia Panel[75,76,102,103] developed several clinical practice guidelines specifically for physical therapists, physiatrists, orthopedic surgeons, rheumatologists, family physicians, and neurologists. These guidelines focused on selected rehabilitation interventions in the management of patients with knee pain, shoulder pain, neck pain, and LBP using rigorous methodology. The panel concluded that the evidence related to the use of massage in patients with these disorders is insufficient, and they were unable to include or exclude therapeutic massage alone as a recommended intervention.

Deep transverse friction massage, a manual treatment technique often used in the management of patients with tendonitis, was the focus of a Cochrane review[73]. This technique is proposed to decrease pain secondary to the reduction in adhesions of the tendon, with increased mobility and realignment of the connective tissue. The two studies that met review criteria combined deep transverse friction massage with other physical therapy modalities. This specific massage technique combined with additional modalities was found not to reduce tendonitis symptoms significantly in the reviewed studies.

## Nerve Mobilization

Recently, the manual application of forces for the purpose of increasing nerve mobility has gained popularity. Incorporating concepts of adverse neural mechanical tension in nerves[104], manual techniques to assess and improve nerve mobility and transmission of tension have been developed[105,106,107]. The ability of nerves to slide relative to other tissues and elongate have been demonstrated with movement[105,106,108,109] and related to impairment[110]. The manual techniques and/or exercises designed to glide or tensilely load specific nerves include sequential positioning of the extremity joints, validated by cadaver studies[111,112]. Limited research exists with these techniques[114,115].

## RESEARCH

Manual therapy is used extensively in the management of patients with musculoskeletal disorders. The inclusion of manual therapy in multiple national clinical practice guidelines attests to that acceptance of the importance of the role of manual therapy. The current demand in health care for evidence-based practice behooves a movement towards more specificity in the research of the effectiveness of manual therapy, with emphasis on specific patient signs and symptoms, and specific manual techniques that result in effective care. The generic approach seen in the research of the role of spinal manipulation has led to confusion and limited extrapolation. Subgroups of patients possibly exist that may respond differently to specific forms of manual therapy. Identification of these subgroups according to patient history and physical examination findings is needed[45,46,47,49,50,51,53,54,55]. In addition to greater attention to specific patient characteristics that may influence treatment effectiveness, future research is needed to give more consideration for specific treatment parameters. Well-designed studies that investigate the role of specific parameters, such as force direction, frequency, duration, speed, and intensity, are needed to clarify the future role of manual therapy in the management of patients with various disorders.

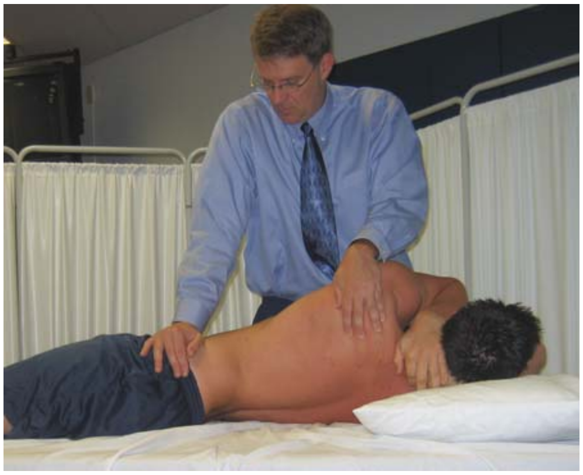

**FIGURE 1.** Technique of manipulation of lumbosacral region used in studies by Childs et al.[53] and Flynn et al.[54].

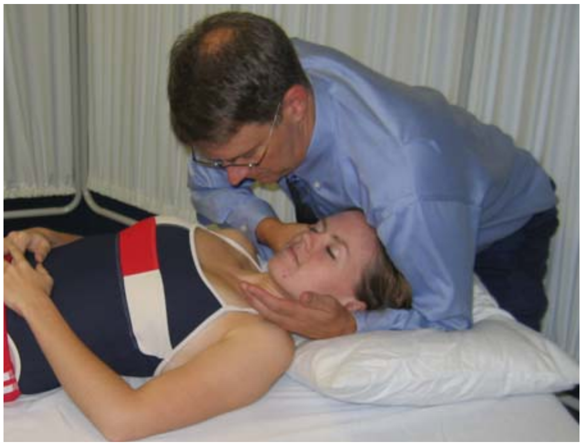

**FIGURE 2.** Cervical manipulation into sidebending.

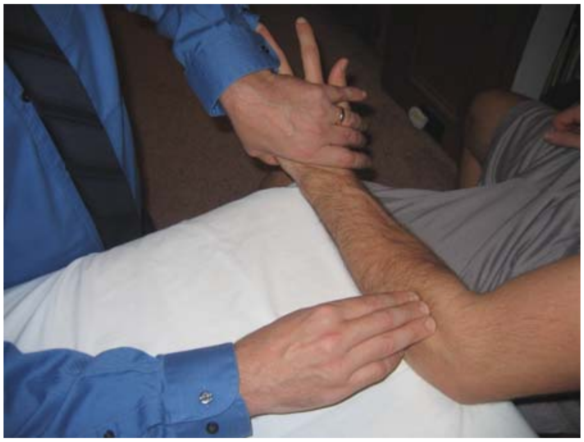

**FIGURE 3.** Example of transverse friction massage to wrist extensors.

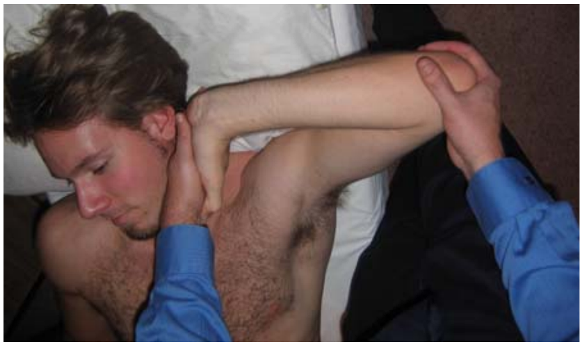

**FIGURE 4.** Neural tension manipulation of ulnar nerve.

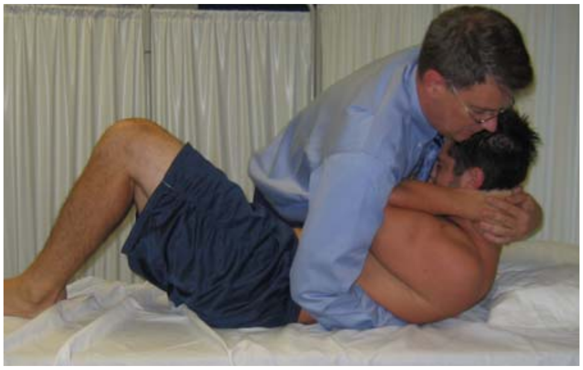

**FIGURE 5.** Manipulation to the mid-thoracic spine.

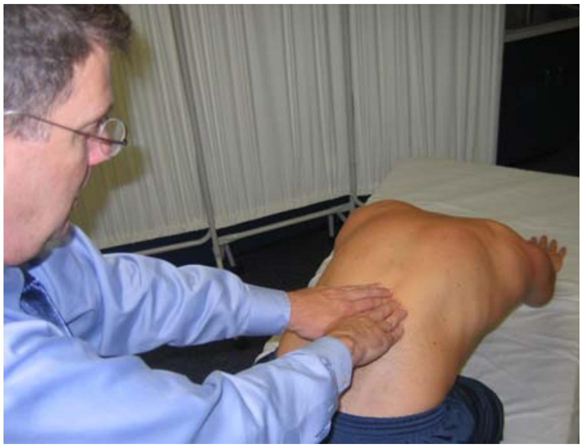

**FIGURE 6.** Massage to the lumbar spine combined with stretching.
